# Effect of Dietary α-Ketoglutarate Supplementation on the Performance, Gut Health, Gene Expression, Antioxidant Capacity, and Hematology in Broilers

**DOI:** 10.3390/vetsci13050470

**Published:** 2026-05-13

**Authors:** Hagar Elashry, Husam H. Nafea, Ahmed Khalid Ahmed, Noor Naji Alhayani, Mostafa Elashry, Rania Elsayed Mahmoud, Tarek Ibrahim, Abeer Aziza, Mutassim Mohamed Abdelrahman

**Affiliations:** 1 Department of Nutrition and Clinical Nutrition, Faculty of Veterinary Medicine, Mansoura University, Mansoura 35516, Egypt; ranianutrition@yahoo.com (R.E.M.); tarrik67@yahoo.com (T.I.); 2 Department of Animal Production, Faculty of Agriculture, University of Anbar, Anbar 55431, Iraq; ag.husam.nafea@uoanbar.edu; 3 Department of Animal Production, College of Agriculture, Tikrit University, Tikrit 34001, Iraq; ahmedkhalid76700@tu.edu.iq; 4 Department of Animal Genetics, Breeding, and Reproduction, College of Animal Science and Technology, Northeast Agricultural University, Harbin 150030, China; 5 Department of Microbiology, College of Medicine, University of Anbar, Anbar 55431, Iraq; med.noor.naji@uoanbar.edu.iq; 6 Department of Animal Production, Faculty of Agriculture, Mansoura University, Mansoura 35516, Egypt; elashry2000@mans.edu.eg; 7 Key Laboratory of Dairy Cow Genetic Improvement and Milk Quality Research of Zhejiang Province, College of Animal Sciences, Zhejiang University, Hangzhou 310058, China; 8 Department of Animal Production, College of Food and Agricultural Sciences, King Saud University, Riyadh 11451, Saudi Arabia; amutassim@ksu.edu.sa

**Keywords:** AKG, blood indicator, broiler, histology, growth performance

## Abstract

Background: Due to α-Ketoglutaric acid (AKG)’s anti-inflammatory and antioxidant properties, it may serve as a beneficial feed supplement for broilers. This study aimed to evaluate the impact of AKG supplementation in broiler diets on growth efficiency, blood indicators, and histological status. Methods: Two hundred sixteen unsexed, one-day-old broilers were randomly assigned to three experimental groups utilizing a completely randomized design. Each treatment was then subdivided into six replicates, each consisting of twelve chicks. In the experiment, the 1st group received a control (basal diet); the 2nd group received the basic diet supplemented with 0.5% AKG; and the 3rd group received the basal diet supplemented with 1.0% AKG. Results: The findings indicate that the addition of 0.5% and 1% AKG to broiler feed significantly (*p* < 0.05) improved BW, BWG, and FCR, particularly during the early stages of growth. The 0.5% AKG group had better feed efficiency and less FI, which means they grew faster and used nutrients better. AKG administration significantly (*p* < 0.05) increased TP and albumin levels in avian subjects, while simultaneously decreasing MDA and elevating CAT concentrations. Adding AKG to the broiler diet raises the levels of RBC, Hb, and PCV, but lowers RDW_CV. The results indicate that AKG lowers inflammation by raising IL-10 levels and lowering IL-1β levels. It also raises levels of antioxidant enzymes like SOD and CAT. Microscopic analysis revealed normal jejunal mucosa, submucosa, and muscular layers across all groups, including those receiving AKG supplements. The jejunal villus height, crypt depth, and their ratio were unchanged. The structure and function of the intestinal lining showed no significant changes when compared to the control. Conclusions: Adding AKG to broiler feed helps their growth, biochemical, and immune system indicators, but it does not hurt their histological conditions.

## Simple Summary

This study investigates the potential application of α-ketoglutaric acid (AKG) as a dietary supplement for broilers to promote growth and maintain health amid challenges in poultry production. AKG has properties that fight inflammation and free radicals. The study aimed to evaluate the effects of AKG on growth efficiency, blood parameters, and intestinal histology in 216 one-day-old broilers, categorized into three groups: a control basal diet, 0.5% AKG, or 1.0% AKG, with six replicates of 12 chicks each. Results showed that body weight, weight gain, and feed conversion ratio (especially at 0.5% AKG, which also led to less feed intake) all improved significantly. Levels of total protein, albumin, RBC, Hb, PCV, IL-10, SOD, and CAT all went up, while levels of MDA, RDW_CV, and IL-1β all went down. Jejunal histology remained normal with no changes in villus height, crypt depth, or ratio. In conclusion, AKG supplementation boosts broiler growth, biochemical, and immune parameters without histological harm. This provides societal benefits by enhancing poultry productivity, improving feed efficiency, and promoting animal welfare. It has the potential to lower costs and support sustainable food security in animal agriculture.

## 1. Introduction

Over the last 50 years, the broiler sector has seen remarkable advancements—including a 116% increase in average slaughter weight and a 38–50% improvement in feed conversion ratio—that are critical to meeting the escalating demand for animal-based protein amid a global population projected to surpass 10 billion by 2050, while maintaining environmental sustainability [1,2]. However, this intensive genetic selection for rapid growth and high feed intake under modern high-density breeding conditions often forces birds to operate near their metabolic limits. Consequently, modern broilers frequently suffer from chronic oxidative stress and subclinical intestinal inflammation, which can impair nutrient absorption and overall health [3,4,5]. Poultry products, especially from broilers, represent a vital source of animal protein [6,7]. These birds dominate multiple industries owing to their economic viability, short production cycle, and high nutritional value [8,9]. However, conventional poultry production systems confront intensifying ecological and economic pressures amid rising chicken output, underscoring the need to enhance broiler growth performance, health status, and environmental sustainability to fulfill consumer demands for healthier products [10,11]. Conventional antibiotics for chicken growth have bred resistant bacteria, threatened public health, and necessitated safe natural alternatives like plant products, herbs, prebiotics, probiotics, and organic acids [12,13].

Alpha-ketoglutaric acid, sometimes referred to as 2-oxoglutaric acid or 2-oxopentanedioic acid, is present in all living creatures and serves as a crucial low-molecular-mass molecule within cells [14]. AKG, the anion of α-ketoglutaric acid, acts as a key intermediary in the tricarboxylic acid (TCA) cycle and nitrogen and amino acid metabolism [15]. In cells, AKG is primarily generated via the oxidative decarboxylation of isocitrate in the TCA cycle and the oxidative deamination of glutamate. As a TCA cycle intermediate, AKG fuels ATP synthesis through mitochondrial oxidative phosphorylation and serves as a substrate for glutamate dehydrogenase and amino acid transferases, supporting amino acid and protein synthesis [16]. Antibiotics in poultry are criticized for resistance, microbiota disruption, and residues. Mainstream natural alternatives—plant extracts (inconsistent bioavailability, costly, and toxic), organic acids (unstable, limited action), and probiotics/prebiotics (strain-specific, poor survivability, variable, and expensive)—also have significant limitations [11,13]. In contrast, AKG stands out as an endogenous metabolite with multifaceted advantages that address these shortcomings. Beyond its core roles in energy metabolism, AKG exerts potent antioxidant capacity by scavenging reactive oxygen species and replenishing glutathione, anti-inflammatory effects via NF-κB pathway inhibition, and intestinal protection through enhanced barrier integrity and mucin production [14]. Uniquely, AKG improves nitrogen utilization efficiency by facilitating glutamate synthesis and transamination, thereby reducing urinary nitrogen excretion, minimizing livestock waste emissions, and lowering overall feed consumption [15]. These attributes position AKG as a stable, cost-effective, and holistically efficacious novel substitute for antibiotics, underscoring the necessity and innovation of this study in advancing sustainable animal nutrition. AKG is highly soluble in water and acts as a precursor to glutamine without causing any negative side effects [17]. Dietary AKG supplementation has demonstrated enhancements in skeletal development in laying hens [18], intestinal metabolic rate and antioxidative capability in ducks [19], and meat quality in laying chickens [18]. AKG has demonstrated the capacity to diminish oxidative stress, stimulate the antioxidant system, and enhance the resilience of S. cerevisiae cells to hydrogen peroxide [20]. In grass carp, AKG treatment resulted in a marked enhancement of glutathione peroxidase 1 (GPX1), CAT, and superoxide dismutase activities, concurrently decreasing the MDA concentration in blood [21].

To our knowledge, there are a few studies on the potential effects of giving AKG to broiler chickens. The rationale for including gene expression analysis was to elucidate the molecular mechanisms underlying AKG’s phenotypic effects. We hypothesized that AKG supplementation would: (1) upregulate hepatic IGF-1 expression, reflecting enhanced growth; (2) upregulate hepatic SOD1 expression as part of an improved antioxidant defense; and (3) upregulate intestinal IL-10 expression and downregulate IL-1β expression, indicating an anti-inflammatory response. This study aimed to investigate the effects of adding AKG on antioxidant activity, gene expression related to antioxidants in the liver, growth efficiency, and blood chemistry in broilers.

## 2. Materials and Methods

### 2.1. Experimental Design, Birds, and Diets

A total of 216 one-day-old, unsexed Cobb 500 broiler chicks obtained from a local commercial hatchery (Mansoura, Egypt) with a similar initial body weight (BW) of 44.35 ± 0.16 g were divided into 3 experimental groups using a completely randomized method. Chicks were not sexed because using unsexed birds better reflects real-world farming conditions, where sex separation is often not performed. Chicks were housed in cages measuring 100 × 100 × 50 cm (length × width × height). Stocking density was 12 birds per cage, equivalent to 0.083 m^2^/bird (or 48 kg/m^2^ based on final BW). Each treatment was divided into 6 replicates, each consisting of 12 unsexed chicks. The trial went on for six weeks. The fundamental diet was formulated based on National Research Council [22] standards and provided in pellet form throughout the research. The diet was structured into 3 phases: the 1st phase (1–15 days), the 2nd phase (15–30 days), and the 3rd phase (30–42 days), as outlined in Table 1. The three dietary interventions were as follows: The control group received a standard diet, the second group received a standard diet augmented with 0.5% AKG, and the third group received a standard diet augmented with 1.0% AKG.

The experiment was conducted under control of environmental conditions. During the 6-week rearing period, ambient temperature gradually decreased from 33 ± 1 °C on day 1 to 22 ± 1 °C by day 28 and maintained thereafter, following standard broiler management guidelines. Relative humidity was maintained at 55–65% throughout the experiment. A 23L:1D lighting schedule (23 h of light and 1 h of darkness) was applied for the entire period, with a light intensity of approximately 20 lux during the first week and 5 lux thereafter. Ventilation was provided continuously using exhaust fans to maintain air quality (ammonia level < 10 ppm) and ensure proper air exchange (0.5–1.0 m^3^/h per kg live weight). All birds had ad libitum access to feed and fresh water via nipple drinkers and trough feeders.

### 2.2. Growth Performance

Each week, every bird was weighed separately. Their BW was documented utilizing a digital balance scale to the nearest 0.1 g. Body weight gain (BWG) was evaluated weekly from 1 to 42 days of age. This procedure was executed regularly at the same time each week. BWG is the final BW minus the initial BW at the start of each period. Feed intake (FI) was calculated by deducting the leftover feed from the provided quantity. The FCR was determined by the g of feed ingested per unit of BWG.

### 2.3. Blood Parameters

For all sample collections (blood, tissues, and histology), the same birds per treatment were used to maintain consistency across analyses. At 42 days of age, following a 6 h fasting period, samples of blood were collected from the wing veins of six birds per treatment immediately before slaughter. Blood was drawn into two separate tubes using sterile 22-gauge needles and 5 mL syringes. “White blood cell (WBC)” counts were evaluated in fresh sanguine specimens using a NIHON KOHDEN automated hematology analyzer, the manufacturer of the Nihon Kohden automated hematology analyzer (Model MEK-6550, Tokyo, Japan). The initial sample was collected in vacuum tubes containing lithium heparin (anticoagulant concentration: 15 IU/mL blood) for hematological analysis. These samples were kept on ice and analyzed within 4 h of collection, encompassing the assessment of red blood cells, hemoglobin (Hb), and packed cell volume (PCV). The mean corpuscular volume (MCV), mean corpuscular hemoglobin (MCH), and mean corpuscular hemoglobin concentration (MCHC) were calculated utilizing the methodologies outlined by Harvey [22]. A second sample was collected in a non-heparinized tube. Blood was allowed to clot at room temperature (22–25 °C) for 30 min. The serum was separated and centrifuged at 3000 rpm for 15 min. The separated serum was aliquoted into sterile Eppendorf tubes and stored at −20 °C for a maximum of 2 weeks until analysis. The serum was analyzed for total protein, albumin, total cholesterol, and creatinine, as well as liver enzyme activity, specifically ALT and AST levels, according to the methodology of Young and Friedman [23] using commercially available kits (Bio-diagnostic, Dokki, Giza, Egypt).

### 2.4. Antioxidant and Digestive Enzymes

After slaughter, tissue samples were collected from the left lateral lobe of the liver and the mid-jejunum (from the segment between the pancreatic loop and Meckel’s diverticulum). Samples were immediately washed with ice-cold phosphate-buffered saline (PBS) to remove blood and intestinal contents, blotted dry, and processed as described below. Approximately 0.5 g of each tissue sample was weighed and minced with scissors on an ice-cold glass plate. The minced tissue was homogenized in 5 mL (1:10 *w*/*v*) of ice-cold homogenization buffer consisting of 50 mM Tris-HCl, 150 mM KCl, 1 mM EDTA, and 0.5% Triton X-100, pH 7.4 (adjusted with HCl/NaOH). Homogenization was performed using a mechanical homogenizer (Teflon-glass pestle) at 1500 rpm with 10 strokes while keeping the tube immersed in an ice-water bath throughout the procedure to prevent protein degradation.

The homogenates were centrifuged at 3000× *g* for 15 min at 4 °C. The resulting supernatants were carefully collected (avoiding the lipid layer and pellet) and aliquoted into sterile microcentrifuge tubes. Supernatants were stored at −80 °C for a maximum of 1 month (for antioxidant enzymes) or at −20 °C for a maximum of 5 days (for digestive enzyme activity assays) until analysis. Repeated freeze–thaw cycles were avoided. Catalase (CAT) and malondialdehyde (MDA) were assessed in liver supernatants, while lipase and amylase activities were measured in jejunal supernatants using enzymatic colorimetric methods with commercial kits (Bio-diagnostic, Dokki, Giza, Egypt) according to the manufacturer’s instructions [24]. All assays were performed in duplicate.

### 2.5. Histological Status

At the end of the experiment (42 days of age), 6 birds were randomly selected from each group (*n* = 6 per treatment) for histomorphological examination. Tissue samples (approximately 1 cm in length) were collected from the mid-jejunum (the same segment between the pancreatic loop and Meckel’s diverticulum as described in Section 2.4). Samples were gently flushed with ice-cold phosphate-buffered saline (PBS, pH 7.4) to remove intestinal contents, then immediately placed in 10% neutral buffered formalin (NBF). The fixation duration was 48 h at room temperature (22–25 °C), with one change in fresh fixative after 24 h.

After fixation, tissue samples were rinsed in running tap water for 30 min, then subjected to a standardized automated tissue processing protocol using a tissue processor (Leica TP1020, Leica Biosystems, Nussloch, Germany). The processing schedule was as follows: 70% ethanol for 1 h, 80% ethanol for 1 h, 90% ethanol for 1 h, 95% ethanol for 1 h, and three changes of absolute ethanol (100%) for 1 h each. Clearing (xylene): three changes of xylene for 45 min each. Paraffin infiltration: three changes of molten paraffin wax (melting point 56–58 °C) for 1 h each.

After infiltration, tissues were embedded in paraffin blocks using an embedding station (Leica EG1150H, Leica Biosystems). Tissue sections were cut at a thickness of 5 μm using a rotary microtome (Leica RM2255, Leica Biosystems). The cutting orientation was transverse/perpendicular to the longitudinal axis of the jejunum to ensure vertically oriented villi (full-length villi from tip to base). Sections were floated in a warm water bath (40 °C), mounted on positively charged glass slides (Superfrost Plus, Thermo Fisher Scientific, Waltham, MA, USA), and dried overnight at 37 °C.

Sections were deparaffinized in xylene (two changes, 5 min each), rehydrated through a descending ethanol series (100%, 95%, 70%, 50%; 3 min each), and stained with hematoxylin and eosin (H&E) according to the standard protocol described by Fossati et al. [25]. Histological slides were examined under an Olympus BX41 light microscope (Olympus, New York, NY, USA) equipped with a DVC 1300C color digital camera. For each bird, three non-overlapping, well-oriented fields were examined at 40× and 100× magnification. The following parameters were measured using ImageJ software (version 1.53, National Institutes of Health, Bethesda, MD, USA, http://imagej.nih.gov/ij/22/3/2025 accessed on 26 April 2026) according to the method of Bancroft and Gamble [26,27]: Villus height (VH, μm): measured from the villus tip to the villus–crypt junction. Crypt depth (CD, μm): measured from the villus-crypt junction to the base of the crypt. Villus height to crypt depth ratio (VH/CD) was calculated.

For each bird, a minimum of 10 intact, vertically oriented villi and their associated crypts were measured (total of 30 measurements per bird across the three fields). Only villi showing intact epithelium from tip to base with clearly visible crypts were included in the analysis. All morphometric measurements were performed by a pathologist who was blind to the treatment allocations.

### 2.6. Real-Time Polymerase Chain Reaction (RT-PCR)

Total RNA was extracted from 0.5 g of liver tissue (for *IGF-1* and SOD1) and 0.5 g of jejunal tissue (for *IL-10* and *IL-1β*) using the RNeasy Mini Kit (Qiagen, Hilden, Germany) according to the manufacturer’s instructions. RNA concentration and purity were assessed using a Thermo Scientific NanoDrop 2000 spectrophotometer (Thermo Fisher Scientific, Waltham, MA, USA). RNA purity was verified by measuring the A260/A280 ratio, with an acceptable range of 1.8–2.0. Additionally, the A260/A230 ratio was assessed to detect protein or organic contamination, with values >2.0 considered acceptable. RNA integrity was confirmed by 1% agarose gel electrophoresis, showing clear 28S and 18S ribosomal RNA bands without smearing.

First-strand cDNA was synthesized from 1 μg of total RNA using the High-Capacity cDNA Reverse Transcription Kit (Applied Biosystems, Foster City, CA, USA). The reverse transcription reaction mixture (20 μL total volume) consisted of 2 μL 10× RT buffer, 0.8 μL 25× dNTP mix (100 mM), 2 μL 10× RT random primers, 1 μL MultiScribe™ Reverse Transcriptase (50 U/μL), 1 μL RNase inhibitor (20 U/μL), 1 μg RNA template, and nuclease-free water to 20 μL. The reaction was carried out in a thermal cycler (Bio-Rad T100, Hercules, CA, USA) under the following conditions: 25 °C for 10 min, 37 °C for 120 min, and 85 °C for 5 min to inactivate the enzyme. The resulting cDNA was stored at −20 °C until use.

All primers used in this study are listed in Table 2. Before qPCR analysis, amplification efficiency (E) was determined for each primer pair using a 10-fold serial dilution of pooled cDNA (5 points, ranging from 1 ng to 1000 ng). Efficiency was calculated according to the formula: E = (10^^(−1/slope)^ − 1) × 100%. Only primer pairs with efficiency between 90% and 110% and a correlation coefficient (R^2^) > 0.98 were used for subsequent analysis. The specificity of each primer pair was confirmed by melting curve analysis (single peak) and by agarose gel electrophoresis (single band at the expected size). The expression stability of two reference genes, β-actin and 28S rRNA, was evaluated under our experimental conditions using the geNorm algorithm (Biogazelle, Zwijnaarde, Belgium). The stability value (M-value) was calculated, and genes with M < 0.5 were considered stably expressed. Both β-actin and 28S rRNA showed M-values below 0.5, indicating suitable stability. The geometric meaning of both reference genes was used for normalization of target gene expression.

The qPCR was performed using the Agilent MX3005P (v4.0) Real-Time PCR System (Agilent Technologies, Santa Clara, CA, USA). Each reaction (20 μL total volume) contained: 10 μL of 2× SYBR Green Premix Ex Taq™ (Tli RNaseH Plus, Takara Bio, Kusatsu, Japan), 0.4 μL of 10 μM forward primer, 0.4 μL of 10 μM reverse primer, 2 μL of cDNA template (diluted 1:5 with nuclease-free water), 0.4 μL of 50× ROX reference dye, and 6.8 μL of nuclease-free water. The amplification program was as follows: initial denaturation at 95 °C for 30 s, followed by 40 cycles of 95 °C for 5 s (denaturation) and 60 °C for 30 s (annealing/extension). Melting curve analysis was performed immediately after amplification under the following conditions: 95 °C for 15 s, 60 °C for 1 min, followed by a gradual temperature increase from 60 °C to 95 °C at 0.3 °C/s with continuous fluorescence acquisition. All reactions were run in triplicate for each sample, and no-template controls (NTCs) were included in each run to rule out contamination.

The threshold cycle (Ct) values were determined using the Agilent MX3005P software. The relative expression of target genes was calculated using the 2^^(−ΔΔCt)^ method as described by Yuan et al. [28], after normalization to the geometric mean of the reference genes (β-actin and 28S rRNA). The control group was used as the calibrator (assigned a value of 1). Results are presented as mean fold-change ± SEM.

**Table 2:** The AKG primer sequence for RT-qPCR.

Gene	Primer Sequence (5′-3′)	Reference
*IL-1β*	GCTCTACATGTCGTGTGTGATGAG	[29]
	TGTCGATGTCCCGCATGA	
	(FAM) CCACACTGCAGCTGGAGGAAGCC (TAMRA)	
*28S rRNA*	GGCGAAGCCAGAGGAAACT	[30]
	GACGACCGATTTGCACGTC	
	(FAM) AGGACCGCTACGGACCTCCACCA (TAMRA)	
*IL-10*	CATGCTGCTGGGCCTGAA	
	CGTCTCCTTGATCTGCTTGATG	
	(FAM) CGACGATGCGGCGCTGTCA (TAMRA)	
*β-actin*	CCACCGCAAATGCTTCTAAAC	[31]
	AAGACTGCTGCTGACACCTTC	
*SOD1*	AGGGGGTCATCCACTTCC	[32]
	CCCATTTGTGTTGTCTCCAA	
*IGF-1*	CAGAGCAGATAGAGCCTGCG	[33]
	TCTGCAGATGGCACATTCAT	

### 2.7. Statistical Analysis

Data were analyzed using one-way analysis via the general linear model procedure in SPSS (version 27.0). Normality was assessed using the Shapiro–Wilk test, and homogeneity of variances was verified with Levene’s test; all assumptions were met (*p* > 0.05). Post hoc differences among treatments were determined using Duncan’s multiple range test. Orthogonal contrasts evaluated linear and quadratic effects of dosage levels on response parameters. Differences were considered significant at *p* < 0.05 and highly significant at *p* < 0.01. Data are presented as means ± standard error of the mean (M ± SEM). The model was:

Y_ij_ = μ + T_i_ + e_ij_

where Y_ij_ is the observation, μ is the overall mean, T_i_ is the fixed effect of treatment i, and e_ij_ is the random experimental error. Graphs were generated using GraphPad Prism (version 8.0).

## 3. Results

### 3.1. Growth Efficiency

The data in Table 3 shows how adding AKG to the feed of broilers affects their BW, BWG, FI, and FCR. The initial BW of the birds showed no significant differences (*p* = 0.807), indicating the homogeneity of the birds in the trial treatments. Supplementation with 0.5% and 1% AKG enhanced various critical growth performance metrics in broiler chickens relative to the control group across most growth phases. Notable linear elevations (*p* = 0.031) in BW and FBW were recorded, especially during the initial week, with 1% AKG frequently yielding the highest weights among the groups, as seen by linear effects. Body weight gain (BWG) exhibited notable (*p* = 0.031) linear enhancements in the AKG groups during the rapid growth phases (weeks 0–1 and 2–3), signifying improved growth efficiency. Additionally, FI was reduced in the AKG groups, showing statistically significant overall, linear, and quadratic decreases (*p* < 0.001) at different times, especially in the 0.5% AKG group. This result means that the feed was used more efficiently. The FCR was significantly improved in the AKG groups throughout the study, indicating that the birds required less feed for each unit of WG. The 0.5% AKG group showed the most significant overall, linear, and quadratic (*p* < 0.000) effects in the following weeks.

### 3.2. Blood Indicators

Table 4 shows blood serum concentrations of creatinine, TP, cholesterol, ALT, AST, albumin, and alkaline pH. TP and albumin concentrations were substantially higher in effect, overall, and quadratic (*p* < 0.015) in 0.5 AKG compared to the control. Additionally, the Alkaline pH was significantly lower in overall and linear effect (*p* < 0.015) in 0.5 AKG compared to the other group.

Table 5 shows that AKG influenced several hematological traits in broilers at 6 weeks of age. Overall treatment effects were significant for RBC (*p* = 0.057; tendency), Hb (*p* = 0.023), PCV (*p* = 0.018), and RDW_CV (*p* = 0.002), whereas MCV, MCH, MCHC, platelet count, and WBC were not significantly changed. Linear effects were significant only for RBC (*p* = 0.029), PCV (*p* = 0.009), and RDW_CV (*p* = 0.007), showing that RBC and PCV declined as AKG level increased, whereas RDW_CV increased linearly. Quadratic effects were significant for Hb (*p* = 0.011) and RDW_CV (*p* = 0.003), indicating a non-linear response, with the 0.5% AKG group generally showing higher Hb, RBC, and PCV values and the 1.0% AKG group showing the highest RDW_CV.

### 3.3. Antioxidants and Digestive Enzymes

Table 6 indicates that supplementary AKG in broiler diets markedly influenced various antioxidant and digestive enzyme metrics at 6 weeks of age. The activity of CAT was significantly (*p* < 0.05) higher in both AKG-supplemented groups compared to the control, with the highest value observed in the AKG 0.5% group. The concentration of MDA, a marker of oxidative stress, was markedly lowered by AKG supplementation, particularly in the AKG 0.5 and AKG 1.0 groups, signifying diminished oxidative damage. The lipase activity showed no statistically significant differences among the groups. Amylase activity was significantly (*p* < 0.05) higher in the AKG 0.5 group compared to both the control and AKG 1.0 groups, suggesting improved starch digestion at moderate supplementation levels. The table’s *p* values indicate significant linear and quadratic effects for CAT, MDA, and amylase, but not for lipase, thus corroborating the advantageous influence of AKG on broiler antioxidant status and certain digestive enzyme activity.

### 3.4. Real-Time Polymerase Chain Reaction (RT-PCR)

**Figure 1:**
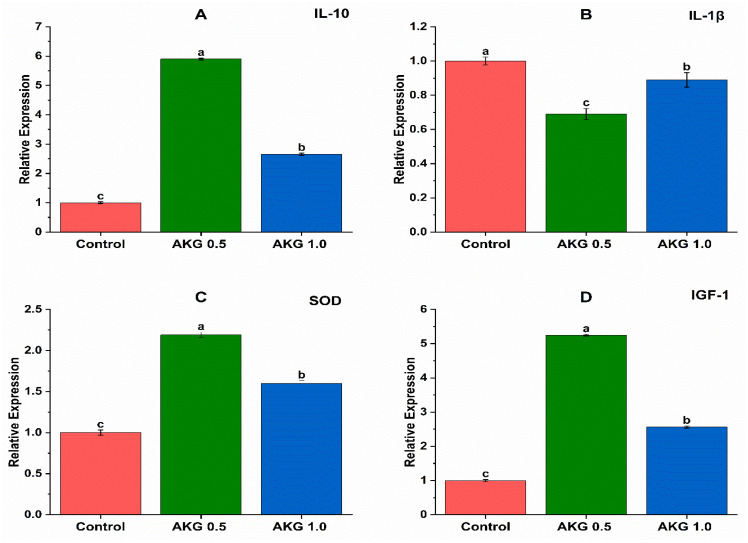
Effect of adding AKG on the antioxidant and anti-inflammatory markers of broilers. (**A**) IL-10; (**B**) IL-1β; (**C**) SOD1; and (**D**) IGF-1. AKG, α-ketoglutaric acid; IL-1β, interleukin-1 beta; IGF-1, insulin-like growth factor-1; SOD1, superoxide dismutase 1; IL-10, interleukin-10. ^a,b,c^ Means within a row followed by different superscripts are significantly different (*p* ≤ 0.05).

As shown in Figure 1A, there were significant differences between all the treatments. AKG 0.5 treatment resulted in increased gene expression of IL-10, followed by AKG 1.0, compared to the control group. Similarly, AKG 0.5 treatment significantly enhanced gene expression of SOD1 and IGF-1, followed by AKG 0.1 compared to the control treatment (Figure 1C,D). Surprisingly, the control treatment induced increased gene expression of IL-1β, followed by AKG 1.0 and AGK 0.5 treatments.

### 3.5. Histological Traits

**Figure 2:**
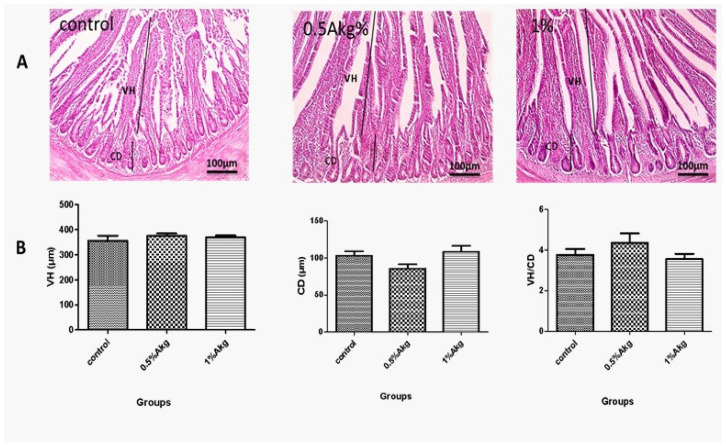
Shows a microscopic examination of the intestine. Microscopic examination showed normal jejunal mucosa, submucosa, and muscular layer in the control group and both supplemented groups (**A**). In the group supplemented with 0.5% AKG, there are non-significantly higher values of jejunal villus height (VH) and villus height to crypt depth ratio (VH/CD), along with lower crypt depth (CD) compared to the other two groups (**B**).

Microscopic examination revealed that the layers of the jejunal mucosa, submucosa, and muscle were normal in all groups, including the control and those that received supplements (Figure 2A). There were no noticeable differences in jejunal VH, CD, or the VH/CD ratio between the control group and the groups that received supplements. As a result, giving AKG supplements did not cause any noticeable changes or problems in the layered structure of the jejunum when compared to the control group. The key measurements of the intestinal lining’s structure and function (VH, CD, and the ratio of VH to CD) did not show any important differences when compared to the control.

## 4. Discussion

Natural nutritional supplements offer practical, cost-effective alternatives for poultry diets, potentially lowering overall production costs [34,35]. However, their effects on chicken production parameters, such as BW and FI, can vary across studies due to differences in dosage, species, and duration [36,37]. In the present study, AKG supplementation optimally regulated broiler growth performance at 1% of the diet, enhancing BW without affecting FI. These findings align with Soltan [38], who reported that broilers fed 1% glutamine—a structurally related amino acid—achieved significantly higher BW compared to controls or groups receiving 0.5%, 1.5%, or 2% glutamine. Excess glutamine (>1%) is similarly depressed BW, mirroring the dosage sensitivity observed here with AKG and underscoring a potential upper threshold for glutamate-family metabolites in broilers. In contrast, lower doses like 0.4 g/kg BW AKG showed only marginal BW effects in turkeys, likely due to species-specific metabolism and shorter trial durations [38,39,40]. Differences in our results versus prior studies on broilers, laying hens, and tilapia—where 1% AKG or glutamine for 56 days had no impact on FI or BW—may stem from physiological divergences: broilers prioritize rapid muscle accretion, amplifying AKG’s benefits, whereas hens focus on egg production with less growth demand [41,42,43]. Mechanistically, AKG drives these growth improvements by acting as a metabolic energy source and potent antioxidant. It bolsters the cellular antioxidant system, reducing oxidative stress and minimizing protein catabolism from body reserves. This spares amino acids for deposition into muscle tissue, enhances liver function and nutrient absorption, and optimizes feed conversion efficiency—forming a complete chain that culminates in superior BW gains in broilers.

Hematological biomarkers reflect livestock’s physiological condition and responses to environmental and nutritional factors [44,45]. Supplementation with 0.5% AKG in the broiler diet significantly improved key blood parameters: RBC count, Hb, and PCV increased, while RDW-CV decreased, with only a minor reduction in WBC count. These changes stem from AKG’s critical roles in cellular metabolism and hematopoiesis. As an integral Krebs cycle intermediate, AKG fuels ATP production, directly supporting the energy demands of bone marrow erythropoiesis [14]. This enhances hematopoietic function, leading to elevated RBC, Hb, and PCV levels, which improve oxygen transport efficiency—a key driver of growth performance, as corroborated by He et al. [46], who linked AKG supplementation to faster growth and better antioxidant status.

The reduction in RDW-CV indicates greater uniformity in RBC size (reduced anisocytosis) [47], reflecting alleviated oxidative stress on erythrocytes. This aligns with findings from Abdel-Moneim et al. [48], where dietary antioxidants mimicking AKG’s properties improved broiler blood profiles under heat stress, and Xanthopoulos et al. [49], who reported a negative correlation between antioxidants and RDW in oxidative stress models. Collectively, these mechanisms underscore AKG’s multifaceted contributions to hematological health, oxygen delivery, and overall broiler productivity.

Supplemental AKG in broiler diets exhibited significant antioxidant properties, as evidenced by increased CAT activity and decreased MDA levels at six weeks of age. These effects stem from AKG’s core molecular mechanism: it acts as a metabolic intermediate that elevates α-ketoglutarate levels, promoting histone demethylation via Jumonji C-domain-containing histone demethylases (JHDMs) and ten-eleven translocation (TET) enzymes. This epigenetic modification enhances Nrf2 transcription and nuclear translocation by reducing repressive histone marks (H3K9me2/3) at the NFE2L2 promoter, thereby upregulating Nrf2 target genes such as CAT, SOD1, and GPX [19,50]. The activated Nrf2 pathway binds antioxidant response elements (AREs) in these gene promoters, boosting their expression and enzymatic ROS-scavenging capacity to alleviate oxidative stress.

In the present study, the dose–response effect showed superior antioxidant outcomes at 0.5% AKG compared to 1.0%, likely due to hormetic biphasic regulation where moderate AKG doses optimally stimulate Nrf2 without inducing feedback inhibition or metabolic overload at higher levels, as observed in other Nrf2-activating nutraceuticals [39,51]. This aligns with the consistent upregulation of SOD1 gene expression paralleling elevated CAT activity, indicating coordinated Nrf2-mediated transcriptional control of the antioxidant enzyme network—SOD1-generated H_2_O_2_ serves as a substrate for CAT, amplifying peroxide detoxification [52]. The significant MDA reduction further confirms AKG’s mitigation of lipid peroxidation, protecting cellular integrity, as corroborated by Wang et al. [51], who reported lowered MDA and enhanced SOD/GSH-Px under heat stress. Overall, these enhancements preserve cellular health and support growth performance under oxidative stress.

The absence of substantial alterations in lipase activity indicates that AKG supplementation does not significantly influence fat-digesting enzymes at the examined dosages. The markedly elevated amylase activity in the 0.5% AKG group compared to both the control and 1.0% groups suggests an optimal dose-dependent influence on carbohydrate digestion. This outcome indicates that modest AKG supplementation improves starch digestion, potentially enhancing food use and energy availability for broilers and mirror carps [50,53]. The reduction in amylase activity at the elevated 1.0% dosage may indicate a limit beyond which additional enzymatic advantages are not realized, underscoring the necessity of dosage optimization in feed additives. These findings underscore the dual function of AKG as both an antioxidant and a digestive modulator in broiler nutrition, aligning with recent studies that highlight its ability to improve antioxidant defenses and digestive enzyme activities, thus promoting enhanced growth and health in poultry [39,50].

A key finding was the dose-dependent upregulation of IL-10 gene expression following AKG supplementation, with the 0.5% dose showing the strongest effect, followed by 1.0%, relative to controls. This aligns with prior studies demonstrating AKG’s enhancement of IL-10, a critical anti-inflammatory cytokine that resolves inflammation and maintains tissue homeostasis [52,54]. Concurrently, AKG at 0.5% markedly increased SOD1 expression—the most pronounced response among doses—bolstering antioxidant defenses against oxidative stress, consistent with reports of AKG upregulating SOD, CAT, and GSH-Px [55,56,57]. AKG also elevated insulin-like growth factor-1 (IGF-1) expression, peaking at 0.5%, promoting anabolic processes and growth performance as seen in livestock models via IGF-1 and growth hormone pathways [58,59].

For pro-inflammatory IL-1β, controls showed higher expression than AKG treatments, where 0.5% and 1.0% doses yielded lower (though still elevated) levels. This pattern suggests AKG mitigates, rather than exacerbates, inflammation. AKG’s anti-inflammatory action centers on inhibiting the NF-κB signaling pathway—a master regulator of pro-inflammatory cytokines, including IL-1β—shifting immune responses toward resolution [58,60]. The relatively higher control IL-1β likely reflects basal inflammation from experimental factors such as housing density, feed transitions, or individual variability, rather than treatment effects. Absolute IL-1β increases across groups may indicate a shared experimental ceiling or mild systemic stress, but AKG’s comparative reduction underscores its protective role without contradicting NF-κB inhibition.

These molecular shifts integrate with phenotypic outcomes: despite potential inflammatory pressures, AKG preserved intestinal barrier integrity (no pathological damage observed), likely via NF-κB-mediated suppression of IL-1β and IL-10 elevation, which collectively enhance epithelial homeostasis and antioxidant protection [52,55,56,57]. Dose dependency, with 0.5% optimal, highlights threshold effects, positioning AKG as an immunomodulatory feed additive for poultry health.

Improvements in growth performance and metabolic efficiency hinge on optimal intestinal structure and function, particularly VH, CD, and the villus-to-crypt (VH:CD) ratio, which collectively determine nutrient absorption capacity and surface area [61,62]. Glutamine, the predominant circulating amino acid, serves as the primary energy source for enterocytes, exerting trophic effects that promote mucosal proliferation, crypt cell regeneration, and epithelial integrity [63].

Histological examination of jejunal samples revealed normal architecture across all groups, including those supplemented with AKG, with no significant differences in VH, CD, or VH: CD ratio compared to controls. This absence of alterations underscores AKG’s inability to disrupt or damage intestinal tissue, affirming its exceptional feeding safety profile—even at supplemental levels. In contrast, prior studies demonstrate glutamine’s more pronounced effects: dietary inclusion of 0.5% glutamine in small mammals elongated villi, deepened crypts, and altered VH: CD ratios [64], while 1% glutamine (10 g/kg feed) enhanced these metrics across intestinal segments versus glutamine-free diets [65]. Notably, 1% glutamine yielded no morphological changes in laying hens’ duodenum, jejunum, or ileum [66].

Elevated VH correlates directly with superior nutrient digestion and absorption [67], while deeper crypts signal accelerated epithelial turnover, often from subtle irritation [68]. A higher VH: CD ratio reflects mature, well-developed mucosa prioritizing absorption over cell renewal, optimizing energy efficiency; lower ratios suggest immaturity, injury, or inflammation [69]. The preserved mucosal, submucosal, and muscular layers in our study indicate no pathological changes such as inflammation, edema, or fibrosis.

Critically, this structural stability with AKG contrasts sharply with many natural feed additives, such as certain phytochemicals (such as high-dose cinnamon or garlic extracts) or herbal mixtures, which frequently induce intestinal inflammation, villus atrophy, and crypt hyperplasia in poultry [70,71]. These additives, while metabolically beneficial, compromise gut integrity and long-term safety, limiting their practical utility in commercial feeds. AKG’s neutral impact thus highlights its superior safety and high efficiency, positioning it as a reliable, non-irritating alternative for enhancing poultry performance without risking enteric damage. This has direct implications for formulating cost-effective, gut-safe diets in intensive production systems.

## 5. Conclusions

Supplementing broiler feed with 0.5% α-Ketoglutaric acid (AKG) markedly improves growth efficiency and fosters general blood health. AKG enhances antioxidant status by activating essential antioxidant enzyme activities and has significant anti-inflammatory properties, evidenced by a substantial rise in the anti-inflammatory cytokine IL-10 and a decrease in the pro-inflammatory cytokine IL-1β. At this concentration, AKG does not induce any detrimental histological alterations or compromise intestinal integrity, underscoring its safety. The data suggest that AKG is a promising natural feed addition for enhancing broiler health and production.

## Figures and Tables

**Table 1:** Composition and calculated analysis of the basal diet.

Feedstuffs	Starter (1–15 Days)	Grower (15–30 Days)	Finisher (30–42 Days)
Ingredients (%) ^1^			
Corn grain	55	61	65.15
SBM	34.75	28.55	24
Corn gluten	4	4	4
Oil	1.9	2.2	2.5
Limestone	1.6	1.6	1.6
Dicalcium phosphate	1.6	1.5	1.6
Premix	0.3	0.3	0.3
Salt	0.3	0.3	0.3
Methionine	0.3	0.25	0.3
Lysine	0.25	0.3	0.25
Total	100	100	100
Calculated analysis ^2^			
CP%	22.9%	20.5%	18.7%
ME (Kcal/kg)	3083	3170	3230
Calcium%	1	0.95	0.95
Available phosphorus%	0.44	0.42	0.41

^1^ Minerals and vitamins premix manufactured by Multi Vita Animal Nutrition^®^ (Tenth of Ramadan City, Sharkia Governorate, Egypt) provides vitamin A 12,000 IU, vitamin D3 2500 IU, vitamin E 20 mg, vitamin K3 2 mg, vitamin B1 2 mg, vitamin B2 5 mg, vitamin B6 2 mg, vitamin B12 0.05 μg, niacin 30 mg, biotin 0.05 μg, folic acid 1 mg, pantothenic acid 10 mg, manganese 60 mg, zinc 50 mg, iron 40 mg, copper 10 mg, iodine 0.6 mg, selenium 0.3 mg per 1 kg diet. DL-methionine (manufactured by Evonik Industries, Essen, Germany) contains 99% methionine. Lysine = lysine hydrochloride (Evonik Industries) and contains 70% Lysine. CP, crude protein; ME, metabolizable energy. Isoenergetic and isoprotein conditions were maintained across all diets by adding AKG at the expense of corn grain without altering CP% or ME values. ^2^ Calculated according to NRC [22].

**Table 3:** Effect of dietary AKG on the growth performance of broiler.

Traits	Age (Weeks)	Treatments			SEM	*p* Value		
		Control	AKG 0.5	AKG 1.0		T	L	Q
BW (g/bird)	0	44.51	44.29	44.24	0.16	0.807	0.543	0.827
	1	117.44 ^b^	119.32 ^ab^	124.15 ^a^	1.12	0.032	0.012	0.478
	2	345.47 ^ab^	349.68 ^a^	335.65 ^b^	2.36	0.034	0.064	0.049
	3	696.47 ^b^	718.86 ^a^	684.59 ^b^	4.07	0.000	0.053	0.000
	4	1097.27	1112.43	1088.89	8.12	0.490	0.674	0.270
	5	1698.08	1731.35	1678.22	19.36	0.212	0.503	0.105
	FBW	2143.36 ^b^	2193.35 ^ab^	2240.62 ^b^	18.40	0.091	0.031	0.970
BWG (g/bird/day)	0–1	72.92 ^b^	75.02 ^b^	79.90 ^a^	1.12	0.023	0.008	0.493
	1–2	228.03 ^a^	230.35 ^a^	211.50 ^b^	2.80	0.004	0.005	0.028
	2–3	350.98 ^b^	369.18 ^a^	348.94 ^b^	3.26	0.010	0.750	0.003
	3–4	400.82	393.56	404.30	7.50	0.853	0.860	0.599
	4–5	600.80	618.92	589.32	6.60	0.188	0.468	0.094
	TBWG	2098.85 ^b^	2149.05 ^ab^	2196.37 ^a^	18.44	0.090	0.031	0.968
FI (g/day)	0–1	210.12	194.77	203.86	3.09	0.122	0.386	0.062
	1–2	606.52 ^a^	512.76 ^c^	542.60 ^b^	9.92	0.000	0.000	0.000
	2–3	653.40 ^a^	532.44 ^b^	629.52 ^a^	14.51	0.000	0.214	0.000
	3–4	783.29 ^a^	647.42 ^b^	748.57 ^a^	19.14	0.003	0.325	0.001
	4–5	962.43 ^a^	813.25 ^c^	905.36 ^b^	15.62	0.000	0.000	0.000
	TFI	3537.28 ^a^	3072.62 ^c^	3313.63 ^b^	51.02	0.000	0.001	0.000
FCR (g feed/g gain)	0–1	2.88 ^a^	2.60 ^b^	2.55 ^b^	0.05	0.014	0.007	0.218
	1–2	2.66 ^a^	2.22 ^b^	2.57 ^a^	0.05	0.000	0.248	0.000
	2–3	1.86 ^a^	1.44 ^b^	1.80 ^a^	0.04	0.000	0.202	0.000
	3–4	1.96 ^a^	1.65 ^b^	1.85 ^a^	0.04	0.006	0.187	0.003
	4–5	1.60 ^a^	1.31 ^c^	1.53 ^b^	0.03	0.000	0.026	0.000
	TFCR	1.68 ^a^	1.43 ^c^	1.51 ^b^	0.02	0.000	0.000	0.000

AKG 0.5, *α*-Ketoglutaric acid 0.5%; AKG 1.0, *α*-Ketoglutaric acid 1.0%; SEM, Standard error of means; ^a,b^ Means within a row followed by different superscripts are significantly different (*p* ≤ 0.05); T, overall effects of treatments; L, linear effects of increasing treatment levels of broiler; Q, quadratic effects of increasing treatment levels of broiler; BW, body weight; BWG, body weight gain; FI, feed intake; FCR, feed conversion ratio.

**Table 4:** Effect of different levels of the AKG on blood constituents of broilers at 6 weeks of age.

Traits	Treatments			SEM	*p* Value		
	Control	AKG 0.5	AKG 1.0		T	L	Q
Creatinine (mg/dL)	0.24	0.25	0.29	0.01	0.563	0.321	0.714
BUN (mg/dL)	0.84	0.83	0.80	0.04	0.949	0.769	0.904
ALT (U/L)	2.66	2.00	2.50	0.21	0.449	0.759	0.226
AST (U/L)	277.66	298.66	317.00	13.41	0.516	0.258	0.964
Alkaline pH	545.33 ^b^	516.50 ^b^	752.00 ^a^	38.55	0.014	0.015	0.062
TP (g/dL)	2.56 ^ab^	2.88 ^a^	2.11 ^b^	0.11	0.014	0.067	0.015
Albumin (g/dL)	2.31 ^ab^	2.60 ^a^	1.98 ^b^	0.10	0.032	0.131	0.025
TC (mg/dL)	128.33	115.50	128.83	3.37	0.194	0.950	0.075

AKG 0.5, *α*-Ketoglutaric acid 0.5%; AKG 1.0, *α*-Ketoglutaric acid 1.0%; SEM, Standard error of means; ^a,b^ Means within a row followed by different superscripts are significantly different (*p* ≤ 0.05); T, overall effects of treatments; L, linear effects of increasing treatment levels of broiler; Q, quadratic effects of increasing treatment levels of broiler; BUN, blood urea nitrogen; ALT, alanine transaminase; AST, aspartate transaminase; TP, total protein; TC, total cholesterol; Alkaline pH, known as basic pH, refers to a solution’s pH value greater than 7.0 on the pH scale, indicating a lower concentration of hydrogen ions (H^+^) and higher concentration of hydroxide ions (OH^−^) compared to pure water. The pH scale ranges from 0 to 14, with 7 considered neutral. Alkaline solutions become more alkaline as the pH approaches.

**Table 5:** Effect of different levels of the AKG on the hematology of broilers at 6 weeks of age.

Traits	Treatments			SEM	*p* Value		
	Control	AKG 0.5	AKG 1.0		T	L	Q
RBC (10^3^/uL)	2.40 ^a^	2.37 ^a^	2.17 ^b^	0.04	0.057	0.029	0.273
Hb (g/dL)	12.18 ^ab^	13.33 ^a^	11.46 ^b^	0.32	0.023	0.188	0.011
PCV%	30.98 ^a^	30.70 ^a^	28.53 ^b^	0.44	0.018	0.009	0.145
MCV (fl)	129.01	129.61	130.95	0.81	0.674	0.405	0.851
MCH (pg)	50.81	56.25	52.63	1.20	0.175	0.502	0.086
MCHC%	39.38	43.41	40.18	0.89	0.148	0.680	0.064
RDW_CV	12.56 ^b^	11.66 ^b^	14.35 ^a^	0.42	0.002	0.007	0.003
Platelet count	14.33	9.83	8.66	1.25	0.145	0.070	0.482
WBC (10^3^/uL)	123.20	114.76	111.38	8.49	0.877	0.633	0.905

AKG 0.5, *α*-Ketoglutaric acid 0.5%; AKG 1.0, *α*-Ketoglutaric acid 1.0%; SEM, Standard error of means; ^a,b^ Means within a row followed by different superscripts are significantly different (*p* ≤ 0.05); T, overall effects of treatments; L, linear effects of increasing treatment levels of broiler; Q, quadratic effects of increasing treatment levels of broiler; RBC, red blood cell; Hb, hemoglobin; PCV, packed cell volume; mean corpuscular volume; MCH, mean corpuscular hemoglobin; MCHC, mean corpuscular hemoglobin concentration; RDW_CV, Red Cell Distribution Width; WBC, white blood cell.

**Table 6:** Effect of different levels of the AKG on antioxidants and digestive enzymes of broilers at 6 weeks of age.

Traits	Treatments			SEM	*p* Value		
	Control	AKG 0.5	AKG 1.0		T	L	Q
CAT (U/mL)	13.29 ^b^	23.62 ^a^	20.65 ^a^	1.39	0.006	0.006	0.008
MDA (nmol-mL)	16.61 ^a^	7.19 ^b^	8.54 ^b^	1.59	0.000	0.000	0.001
Lipase (U/L)	6.83 ^ab^	7.33 ^a^	4.50 ^b^	0.54	0.068	0.069	0.127
Amylase (U/L)	407.50 ^b^	776.16 ^a^	535.33 ^b^	48.28	0.001	0.134	0.001

AKG 0.5, α-Ketoglutaric acid 0.5%; AKG 1.0, α-Ketoglutaric acid 1.0%; SEM, Standard error of means; ^a,b^ Means within a row followed by different superscripts are significantly different (*p* ≤ 0.05); T, overall effects of treatments; L, linear effects of increasing treatment levels of broiler; Q, quadratic effects of increasing treatment levels of broiler; CAT, catalase; MDA, malondialdehyde.

## Data Availability

The data presented in this study are available on request from the corresponding author due to data privacy and ethical constraints related to the confidentiality of animal records and farm-level production data held by the university.

## Abbreviations

The following abbreviations are used in this manuscript:

AKG	α-Ketoglutaric acid
TCA	tricarboxylic acid
GPX1	glutathione peroxidase 1
SOD1	superoxide dismutase 1
IL-10	interleukin-10
IGF-1	insulin-like Growth Factor-1
IL-1β	interleukin-1 beta
CAT	catalyze
MDA	malonaldehyde
BW	body weight
BWG	body weight gain
FI	Feed intake
FCR	Feed conversion ratio
Hb	hemoglobin
PCV	packed cell volume
MCV	mean corpuscular volume
MCHC	mean corpuscular hemoglobin concentration
TP	total protein
TC	total cholesterol
AST	aspartate transaminase
ALT	alanine transaminase
CD	crypt depth
VL	villus height
